# A neuropsychological perspective on sensory hypersensitivity after acquired brain injury

**DOI:** 10.1093/braincomms/fcag199

**Published:** 2026-06-18

**Authors:** Jasmijn O Heijman, Marilien C Marzolla, Hella Thielen, Kerstin Spielmann, Marsh Königs, Céline R Gillebert, Caroline M van Heugten, Nathan van der Stoep, Irene M C Huenges Wajer

**Affiliations:** Department of Experimental Psychology, Helmholtz Institute, Utrecht University, Utrecht, CS 3584, The Netherlands; Bartiméus, Zeist, BA 3700, The Netherlands; Faculty of Psychology and Neuroscience, Maastricht University, Maastricht, ER 6229, The Netherlands; Limburg Brain Injury Centre, Maastricht, ER 6229, The Netherlands; Department Brain & Cognition, Leuven Brain Institute, KU Leuven, Leuven 3000, Belgium; Department of Neuropsychology, Rehabilitation Hospital RevArte, Edegem 2650, Belgium; Bartiméus, Zeist, BA 3700, The Netherlands; Amsterdam Reproduction and Development Research Institute, Amsterdam, AZ 1105, The Netherlands; Department of Pediatrics, Emma Children's Hospital, Amsterdam UMC Location University of Amsterdam, Emma Children's Hospital Amsterdam UMC Follow-Me Program & Emma Neuroscience Group, Amsterdam, AZ 1105, The Netherlands; Department Brain & Cognition, Leuven Brain Institute, KU Leuven, Leuven 3000, Belgium; Faculty of Psychology and Neuroscience, Maastricht University, Maastricht, ER 6229, The Netherlands; Limburg Brain Injury Centre, Maastricht, ER 6229, The Netherlands; Department of Experimental Psychology, Helmholtz Institute, Utrecht University, Utrecht, CS 3584, The Netherlands; Human Machine Teaming, Defense, Safety, and Security, TNO, SoesterbergDE 3769, The Netherlands; Department of Medical Psychology, Amsterdam UMC, Amsterdam, HV 1081, The Netherlands

**Keywords:** acquired brain injury, sensory hypersensitivity, neuropsychology, neurocognition, sensory overload

## Abstract

Individuals with acquired brain injury frequently report a heightened sensitivity to sensory stimuli, commonly referred to as sensory hypersensitivity, which can significantly disrupt daily life and diminish quality of life. Despite a prevalence of up to 83%, the topic of sensory hypersensitivity after acquired brain injury is largely overlooked in existing research. Consequently, the underlying causes and perpetuating factors of sensory hypersensitivity remain poorly understood, hindering the development of effective, evidence-based treatments. In this review, we explore sensory hypersensitivity following acquired brain injury from a neuropsychological standpoint, offering hypotheses regarding the potential neurocognitive and psychological mechanisms involved. Specifically, we propose several hypotheses considering various aspects of sensory processing including, but not limited to, sensory gating, habituation, working memory, processing speed, anxiety, personality, coping and fatigue. These hypotheses are based on acquired brain injury research where possible but also incorporate findings from related fields. Finally, we introduce a conceptual model that explains sensory hypersensitivity as a mismatch between an individual’s resources (including the proposed neurocognitive and psychological functions) and the demands imposed by their external sensory environment. We identify several possible mechanisms contributing to sensory hypersensitivity after acquired brain injury and consider a range of factors that may vary per individual. With this review, we aim to establish a foundation for hypothesis-driven research into the causes of sensory hypersensitivity post-injury, by setting a research agenda and supporting the development of psychoeducational and rehabilitative strategies to reduce sensory hypersensitivity and enhance quality of life.

## Teaser

Heijman, Marzolla *et al*. report that sensory hypersensitivity is common after acquired brain injury but poorly understood. They propose neurocognitive and psychological mechanisms based on previous studies and conclude that sensory hypersensitivity reflects a mismatch between individual resources and environmental demands, highlighting directions for targeted rehabilitation and future research.

## Graphical Abstract

**Graphical Abstract:**
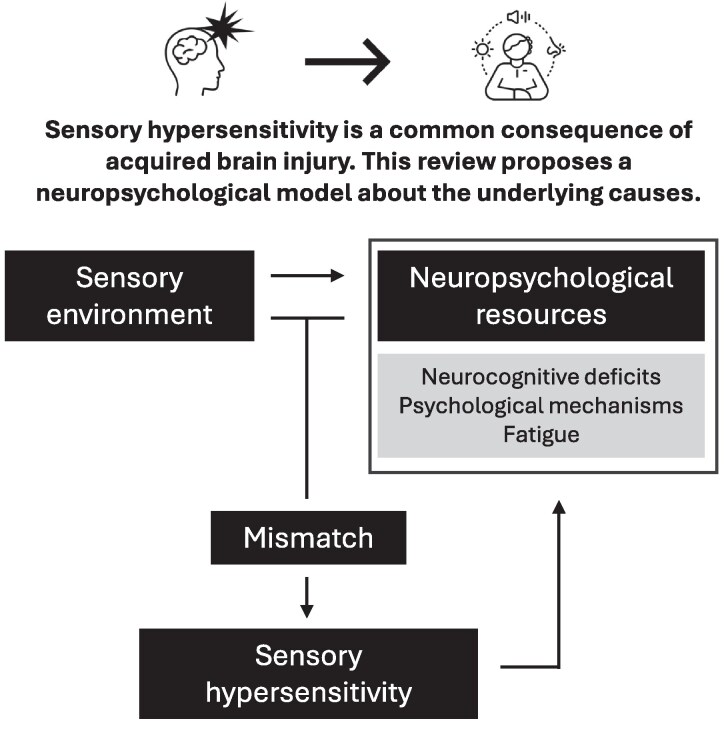

## Introduction

Acquired brain injury (ABI) is a broad term that refers to any brain damage, excluding congenital or degenerative causes. It includes both traumatic injuries as well as non-traumatic causes such as stroke, brain tumours or infections.^1^ Due to the variety in causes and severity, the clinical presentation of ABI is highly diverse. Patients with ABI frequently experience sensory hypersensitivity, with a prevalence of up to 83%.^2^ For these individuals, everyday sensory stimuli such as sunlight or background music can be perceived as very intense or even painful after ABI. As a result, patients often experience fatigue, headaches, or nausea after exposure to stimulus-rich environments^3^ leading to withdrawal, frustration and reduced quality of life.^4^

Likewise, the experience of post-ABI sensory hypersensitivity seems to be heterogeneous. Some ABI patients report feeling overwhelmed by the large number of sensory stimuli in crowded environments such as a shopping mall, a restaurant or at work,^5,6^ whereas other patients’ reports primarily concern the perceived increase in the intensity of single sensory stimuli. Noise and light hypersensitivities are generally most common, but hypersensitivities to visual, tactile, gustatory, olfactory and internal stimuli (e.g. pain and hunger) also occur.^7^ In line with the high interindividual differences regarding the specific experience of sensory hypersensitivity after ABI, the terminology and definitions used vary greatly.^2^ Throughout this review, we will use the term sensory hypersensitivity. A detailed explanation of definitions and terminology can be found in Section 2 (see Table 1).

**Table 1:** An overview of different terms and definitions that are used to describe sensory hypersensitivity within and across different sensory modalities

Across sensory modalities	Within a specific sensory modality	Example of a definition
Sensory hypersensitivity	Hyperacusis/hyperaestesi/hypersomia/hypergeusia Sensitivity to noise/sensitivity to light/tactile, olfactory, gustatory sensitivity	‘A constant, heightened awareness of internal (interoceptive) and/or external (exteroceptive) stimuli’^22^ ‘An over-responsiveness to sensory stimuli’^27^
Sensory processing sensitivity		‘A genetically determined trait involving a deeper cognitive processing of stimuli that is driven by higher emotional reactivity’^8^
Sensory over-responsiveness		‘People with sensory over-responsiveness respond to sensation faster, with more intensity, or for a longer duration than those with typical sensory responsivity’^30^
Sensory overload		‘A perceived increase in the intensity, diversity and/or the pattern of environmental stimuli which exceed the normally experienced level and are thus experienced as aversive’^13^
Sensory intolerance	Sound or noise intolerance/light intolerance/touch intolerance/smell intolerance/taste intolerance	‘A high level of distress evoked by common environmental stimuli across multiple sensory domains’^31^
Sensory flooding		‘A breakdown in selective inhibitory function resulting in flooding by an undifferentiated mass of incoming sensory data’^32^
Sensory defensiveness	Phonophobia, photophobia	‘A tendency to react negatively or with alarm to sensory input which is generally considered harmless or non-irritating’^33^

Sensory hypersensitivity is studied in different populations and scientific fields. In the personality literature, sensory sensitivity has been described as a trait that contributes to individual differences, i.e. sensory processing sensitivity.^8,9^ While sensory hypersensitivity after ABI is sudden-onset and injury-related, sensory processing sensitivity is usually described as a life-long temperament trait.^10^ The current review aims to specifically shed light on sensory hypersensitivity in relation to ABI, where injury-related mechanisms play a key role. Nonetheless, while it is currently unclear how sensory processing sensitivity relates to sensory hypersensitivity after ABI, knowledge from the personality literature can be used to generate hypotheses regarding sensory hypersensitivity after ABI.

Sensory hypersensitivity has also been described in psychiatric conditions like schizophrenia,^11^ attention deficit hyperactivity disorder (ADHD)^12^ and autism spectrum disorder (ASD)^13^ as well as in patients suffering from epilepsy, migraine or chronic pain disorders such as fibromyalgia.^14-16^ Yet even though many people with ABI report sensory hypersensitivity, studies of sensory hypersensitivity after ABI are scarce. Consequently, little is known about the underlying mechanisms of sensory hypersensitivity after ABI, and to date, no evidence-based treatments exist.^2^ A better understanding of sensory hypersensitivity is required for the development of evidence-based treatments, rendering it of great importance to generate testable hypotheses regarding the mechanisms underlying sensory hypersensitivity symptoms after ABI.

This narrative review builds upon the previously published systematic review on sensory sensitivity following ABI.^2^ While their work focused on summarizing the existing findings regarding prevalence and symptom characteristics, the current review adopts a more integrative and theory-driven approach, providing testable hypotheses to guide future research. We first discuss the definition and terminology that are often used to describe sensory hypersensitivity. Next, we discuss previous findings in both ABI and other clinical populations and identify neural, cognitive and psychological mechanisms that may play a role in sensory hypersensitivity after ABI. We end this review by describing a conceptual model that integrates all hypotheses and aims to explain how sensory hypersensitivity may arise from a mismatch between an individual’s resources (e.g. neurocognitive and psychological functions) and situational demands of the (external) sensory environment. This conceptual model is an elaboration on a previously published model by Marzolla *et al*.,^3^ originally based on qualitative data from patient experiences with sensory hypersensitivity following ABI. The simplicity of the model is a benefit for clinical application as it can be used in psychoeducation and for individuals to gain insight in their sensory sensitivity. The current review will expand on this and focus specifically on an explanation and review of the ‘resources’ in the model to dive deeper into the underlying and related mechanisms. With this review, we hope to encourage and inspire future studies on the aetiology of sensory hypersensitivity after ABI and facilitate the development of psychoeducational and rehabilitative approaches.

## Definition and terminology

A variety of terms (sensory processing sensitivity, sensory over-responsiveness, sensory hypersensitivity and sensory overload) is used to describe seemingly similar symptomatology. However, to date it remains unclear whether these terms are synonyms.^10,13,17,18^ Table 1 gives an overview of different terms and definitions that are used to describe sensory hypersensitivity within and across different sensory modalities.

The terms that researchers use in the literature to describe similar symptoms seem to depend on the scientific field and population that is studied. For instance, the term ‘sensory over-responsivity’ (a hyper-reactivity to sensory stimuli) is customary in neurodevelopmental disorders,^19,20^ while in the neurotypical population, similar behaviour is coined as ‘sensory processing sensitivity’.^8^ This lack of standardized terminology limits the exchange of scientific knowledge across fields and populations, complicates compiling an overview of the available literature and impedes evidence-based decision-making. Since the term ‘sensory hypersensitivity’ is used in multiple populations (including the ABI population),^21-25^ we decided to adopt this term in the current review.

In addition to the lack of standardized terminology across populations and fields, there are large differences between the definitions that are used for the same terms. To illustrate, descriptions of sensory hypersensitivity include a heightened awareness of sensory stimuli, being overstimulated by sensory stimuli, a hyperresponsiveness towards sensory stimuli, or feelings of intolerance, anxiety and pain when exposed to sensory stimulation.^13,21,22,26-28^ Furthermore, some definitions (e.g. the definition of sensory processing sensitivity) incorporate empathy and emotional responsiveness in addition to sensory aspects.^29^ To date, there is no consensus on how to define sensory hypersensitivity after ABI. Focusing on the subjective nature of the symptomatology without referring to an underlying mechanism and stressing a change compared to a pre-injury state, we propose the following definition of sensory hypersensitivity complaints after brain injury:

The experience of an increase in the sensitivity to one or multiple extero- or interoceptive sensory stimuli after brain injury as compared to before the brain injury. This increased sensitivity can (but does not necessarily) manifest itself in an altered response towards sensory stimulation (e.g. fatigue, headache, sensory avoidance, irritability during or after sensory stimulation).

## Neural mechanisms

One aspect that differentiates individuals with sensory hypersensitivity after ABI from other populations reporting sensory hypersensitivity is the sudden onset of symptoms, as well as the potential neural changes related to the ABI. Post-injury sensory hypersensitivity may involve several neural mechanisms including (i) structural damage to specific brain regions, (ii) functional abnormalities or (iii) neurotransmitter imbalances.

Hypothesis: Sensory hypersensitivity after ABI is a result of structural brain abnormalities.

A relationship between post-stroke sensory hypersensitivity and lesions in the putamen, thalamus, amygdala and insula was found using multivariate support vector regression lesion-symptom mapping in 103 subacute stroke patients (half of whom reported post-stroke sensory hypersensitivity.^34^ The involvement of these regions in sensory hypersensitivity after ABI can be attributed to various cognitive and psychosocial mechanisms, including selective attention, multisensory integration, working memory and anxiety-related hypervigilance,^35-42^ which are further discussed in this review. In the same study, Thielen *et al*.^34^ did not find evidence for a relationship between lesion volume and the severity of post-stroke sensory hypersensitivity, suggesting that sensory hypersensitivity can occur with similar severity in individuals with mild and severe strokes.

White matter connecting brain regions can also be affected by ABI. Indirect structural disconnection mapping revealed that post-stroke sensory hypersensitivity was associated with damage to the frontal-insular tracts and the fronto-striatal tract.^34^ These tracts play an important role in sensory filtering as well as emotional responses to sensory stimuli as they are involved in the appraisal of sensory stimuli and threat monitoring.^37,43^ Disruptions of these tracts might lead to an overly negative evaluation or inappropriate feelings of threat attributed to neutral sensory stimuli.

Currently, evidence linking sensory hypersensitivity after ABI to structural brain abnormalities is limited. Given the small number of studies, further replication in larger and more diverse samples of brain injury patients is highly recommended.

Hypothesis: Sensory hypersensitivity after ABI is a result of functional brain abnormalities.

Functional magnetic resonance imaging (fMRI) studies in ABI patients with post-injury sensory hypersensitivity are scarce.^2^ Astafiev *et al*.^44^ found that patients with mild traumatic brain injury (mTBI) with light sensitivity showed higher activation in the visual cortex during a visual tracking task compared to those without light sensitivity. Studies in other populations such as neurotypical adults, adults with chronic pain and adults and children with ASD found altered brain activity in the insula, thalamus, amygdala, hippocampus and orbito-frontal cortices in relation to sensory hypersensitivity.^10,11,45,46^ Moreover, research suggests that disrupted connectivity between these brain regions may contribute to sensory hypersensitivity. Green *et al*.^47^ found a relationship between increased connectivity between the thalamus and the amygdala and severity of sensory hypersensitivity symptoms in adolescents with ASD. They propose that this increased connectivity might result in increased attention towards and negative appraisal of irrelevant sensory stimuli.

Beyond functional abnormalities and heightened connectivity between specific regions, recent research has also linked two large brain networks—the salience network and the default mode network—to sensory hypersensitivity.^10,27^ The salience network is involved in the detection of relevant sensory information and filtering of irrelevant information.^48^ Evidence for a relationship between salience network abnormalities and sensory hypersensitivity has previously been found in children with ASD.^49^ The default mode network is representative of a resting condition where the brain disengages from the external world and is focused on internally oriented self-referential activity.^50-52^ Sensory hypersensitivity may arise from a failure of this network to deactivate properly, leading to an excessive focus on external stimuli. Default mode network abnormalities have been reported in clinical groups that report sensory hypersensitivity complaints such as individuals with ASD, ADHD, schizophrenia or chronic pain,^53-56^ but have not been studied in ABI populations.

Hypothesis: Sensory hypersensitivity after ABI is a result of a hyperactive brain as due to neurotransmitter imbalances.

Some research indicates that neurotransmitter imbalances, particularly between the main inhibitory neurotransmitter gamma-aminobutyric acid (GABA) and the excitatory neurotransmitter glutamate, may result in sensory hypersensitivity.^27,57^ Indeed, in children with ASD, elevated glutamate levels in sensorimotor regions and reduced GABA levels in thalamic regions are associated with sensory hypersensitivity.^57,58^ Such an imbalance between GABA and glutamate could lead to increased brain excitability and heightened arousal and attentiveness, potentially explaining the connection to sensory hypersensitivity. While GABA-glutamate imbalances have been observed in brain injury patients,^59,60^ no studies have directly investigated how these imbalances relate to subjective sensory hypersensitivity in this group.

## Neurocognitive mechanisms

There are many cognitive processes that help us deal with the plethora of sensory inputs that we receive and that can be disrupted by ABI. Early in sensory processing, sensory threshold, sensory gating and habituation are of importance, followed by (cognitive) functions such as attention and working memory. To ensure that the processing of the sensory input is efficient, effective multisensory integration and a sufficient processing speed are necessary. These processes aid in preventing the brain’s sensory processing capacity from being exceeded. For each process, hypotheses regarding potential causes of sensory hypersensitivity are discussed.

### Sensory thresholds, gating and habituation

#### Sensory thresholds

Hypothesis: Sensory hypersensitivity after ABI is caused by a lower sensory threshold.

Sensory thresholds, the minimal (change in) stimulus intensity required for detection, are vital in sensory processing. Stimuli exceeding this threshold are further processed and consequently perceived, while weaker stimuli are not. Anecdotally, lower sensory thresholds could result in more stimuli being perceived, which may result in higher sensory sensitivity.

In the literature regarding sensory thresholds in relation to sensory hypersensitivity, Dunn’s^61^ model of sensory processing is often cited. In this model, neurological thresholds and behavioural responses are on a continuum. High and low sensory thresholds are referred to as habituation and sensitization, respectively. The behavioural response continuum consists of responses in accordance with the threshold on the one hand and responses counteracting the threshold on the other hand. According to this model, sensory sensitivity results from low neurological thresholds and a response in accordance with this threshold.^61^ However, the hypothesized neurological thresholds mentioned are measured through a questionnaire, not through objective methods (e.g. EEG, behavioural responses or neuroimaging).

Research on objective sensory thresholds in ABI patients is scarce. A study on post-stroke visual hypersensitivity found lower sensory thresholds compared to stroke patients without visual hypersensitivity.^62^ This study defined thresholds as the shortest exposure duration required to correctly detect a target letter. Additionally, there are two studies investigating the critical flicker fusion frequency threshold in mTBI. Both studies did not find significant differences between people with mTBI and a healthy control group.^63,64^ However, a relationship between subjective light and motion sensitivity and the critical flicker fusion threshold was found in the patient group in one of these studies.^63^ This indicates that the patients with sensory hypersensitivity complaints might not tolerate temporal stimuli as well as they could before the brain injury. The authors suggest that this change in tolerance may be due to neural disinhibition.^63^ This result was not found in a similar study,^64^ possibly due to a difference in reported severity of sensory hypersensitivity.^63,64^

Lastly, sensory thresholds have been studied in ASD populations, but again the number of studies is limited. People with ASD have been found to have a higher visual acuity, for which the authors use the term visual threshold, compared to people without ASD.^65^ This study did, however, not include any measure of visual or sensory hypersensitivity.

#### Sensory gating

Hypothesis: Deficits in sensory gating underlie sensory hypersensitivity after ABI.

Sensory gating is a pre-attentive process (e.g. Wan *et al*.^66^) that has been defined as the phenomenon in which the response to repetitive (irrelevant) stimuli is inhibited.^67^ This keeps higher cognitive functions, such as attention and memory, from being flooded with irrelevant sensory input.^68,69^ It is often measured using an EEG recording with the relevant event-related potential (ERP) occurring 50 ms after sensory stimulation (P50). The operational definition of sensory gating is a diminishing P50 amplitude to a repeated stimulus.^67,70^

Sensory gating deficits have been reported in ABI populations, even though the number of studies is limited. For example, affected auditory ERPs are linked to chronic mTBI.^71^ Specifically, an increase in the P50 amplitude was observed in people who have sustained multiple mTBI compared to people with non-repetitive or no mTBI.^71^ However, whether this increase is related to sensory hypersensitivity was not studied. In another study in people with mild to severe TBI, significantly increased P50 ratios were also observed. The participants in this study reported complaints consistent with impaired auditory gating, such as finding a noisy environment overstimulating.^72^

In other patient populations known to report sensory hypersensitivity, changes in early sensory gating (P50 suppression) have been reported as well. ADHD patients showed less P50 suppression compared to a control group to repeated auditory stimuli.^73^ Reduced auditory gating was also found in migraineurs^74^ and schizophrenia patients (e.g. Magnée *et al*.,^75^ Croft *et al*.^76^; Braff *et al*.^77^). However, these studies did not link sensory gating to sensory hypersensitivity complaints.

#### Habituation

Hypothesis: Sensory hypersensitivity after ABI can be caused by slower habituation.

Habituation is a non-associative learning process in which behavioural responses decrease with repeated exposure to a stimulus.^78^ Sensitization, its counterpart, enhances responses to repeated stimuli^79^ and reflects an enhanced response following repeated administrations of a stimulus. Habituation seems to greatly overlap with sensory gating (Section 4.1.2.). The terms and methods even seem to be used interchangeably across the literature, similar to the terms habituation and sensory thresholds (e.g. Dunn^61^). The methods to measure habituation as a non-associative form of learning specifically differ greatly across studies.^80^

To our knowledge, no studies have examined the relationships between habituation, sensory hypersensitivity and ABI. However, habituation deficits have been linked to sensory hypersensitivity in neuropsychiatric disorders across different sensory domains.^80^ In ASD, habituation and sensory hypersensitivity to mildly aversive auditory and tactile stimuli have been studied using fMRI.^17^ The results were indicative of slower habituation in the amygdala and somatosensory cortex in youth with ASD and sensory hypersensitivity, compared to youth with ASD and without sensory hypersensitivity, or typically developing youth.^17^ Similarly, a correlation between self-reported sensory hypersensitivity and obsessive-compulsive symptoms was found. The participants with high obsessive-compulsive symptoms showed slower habituation, measured via electrodermal activity, compared to participants with low obsessive-compulsive symptoms.^81^ However, findings in other patient populations such as individuals with ADHD are inconsistent (see McDiarmid *et al*.^80^ for a review).

Both sensory gating and habituation have been described as essential in protecting higher-order cognitive functions from ‘overflooding’, by inhibiting (or ‘gating out’) the responses to irrelevant sensory input.^67,68,80^ Similarly, it could be argued that a sensory threshold serves the same purpose. Although the relationship between sensory thresholds, sensory gating, habituation and sensory hypersensitivity after ABI is unknown, it might be possible that sensory gating deficits or a slowdown in habituation after ABI influences cognitive functions such as attention and working memory, which in turn causes the experience of sensory hypersensitivity. This idea is strengthened by the finding that there is a positive relationship between sensory (auditory) gating (P50 suppression) and attentional performance on neuropsychological tests.^66^

### Attention

Attention allows us to select and further process parts of the large amounts of sensory input by focusing on relevant information while ignoring irrelevant information (e.g. Carrasco^82^Carrasco ^82^). However, when problems emerge within this attentional system, it can become increasingly difficult to focus on the right information. We propose that various problems with attentional mechanisms can lead to increased sensory hypersensitivity.

Hypothesis: Sensory hypersensitivity after ABI emerges from deficits in selective attention.

Our capability to successfully select relevant information and focus our attention (i.e. selective attention) may depend on our previous knowledge, goals or the salience of stimuli in the environment. Focusing our attention to a certain part in space can be both goal-driven (i.e. top-down or endogenous attention) or driven by stimuli in the environment (i.e. bottom-up or exogenous attention; e.g. Macaluso and Doricchi^83^; MacLean *et al*.^84^; Carrasco^82^)

Studies regarding ABI and sensory hypersensitivity yield mixed results. In a post-stroke population, results indicated a relationship between impaired selective attention, as measured with an experimental behavioural task based on the Theory of Visual Attention, and visual hypersensitivity.^62^ However, in TBI patients, selective attention, as measured with the computerized CNS Vital Signs neurocognitive testing battery, was not related to noise sensitivity.^85^

Deficits in selective attentional processes have been linked to sensory hypersensitivity in other patient populations. For example, in schizophrenia patients, poor filtering of task-irrelevant auditory stimuli was observed and linked to sensory hypersensitivity (i.e. sensory flooding; see Luck *et al*.^86^ for a review). The authors note that many of the effects discussed in their review could also be explained by impaired attentional control processes, in which (selective) attention is successfully directed but to irrelevant stimuli.^86^

Hypothesis: Sensory hypersensitivity after ABI emerges from a hyperfocus on irrelevant stimuli.

Hyperfocus reflects a state of narrow and heightened attention for a specific aspect in the environment. It can be beneficial, e.g. in ADHD,^87^ but problematic when directed at irrelevant stimuli.^86^ In schizophrenia patients, it has been concluded that a combination of directing attention to irrelevant stimuli and then hyperfocusing on them may explain some of their symptoms. These symptoms include ‘sensory flooding’.^86^ Studies regarding ABI and hyperfocus are, however, very scarce. Only one study reported that in half of the included stroke patients who reported sensory hypersensitivity, a tendency to hyperfocus on irrelevant distractors while ignoring relevant information was observed.^62^

### Working memory

Hypothesis: Sensory hypersensitivity after ABI is caused by a smaller working memory capacity.

Working memory temporarily stores sensory information to guide behaviour^88^ and is necessary to keep things in mind for a short period of time while performing complex tasks.^89,90^ To protect working memory from overload, attention seems to be used as a ‘gatekeeper’.^91,92^ If sensory input is not attended to, we will most likely not become consciously aware of it since it will not have entered working memory.^93^ Working memory in turn plays an important role in top-down control of selective attention by guiding attention to relevant (sensory) information.

The already limited capacity in working memory can be lower in several populations, including patients with ABI.^94,95^ In schizophrenia patients, a reduced working memory capacity was found when compared to healthy controls.^96^ Besides a smaller working memory capacity, it has also been argued that if people fail to filter out irrelevant information, there is less remaining capacity for relevant information.^86^ In neurotypical young adults, filtering ability was indeed correlated to working memory. Filtering ability was determined by comparing brain activity in three brain areas in a distractor versus a no-distractor condition. Overall, the results again stress the importance of both sufficient attentional function and working memory^86,92^ as well as sensory gating and habituation.

Working memory in relation to sensory hypersensitivity has not been studied in ABI populations, but it has in the healthy population. In older healthy adults, high sensitivity to sensory input as measured with the adolescent/adult sensory profile (AASP)^61^ was associated with lower working memory scores as determined using the Behaviour Rating Inventory of Executive Function-Adult Function (BRIEF-A).^97^ Additionally, higher working memory capacity modulates auditory perceptual filtering of irrelevant sound. It was found that an auditory brainstem response decreased as the central working memory load increased, indicative of suppression of irrelevant sounds at early processing stages.^91^ Specifically, auditory brainstem responses decreased as the central working memory load increased, indicative of suppression of irrelevant sounds at early processing stages. Moreover, the authors found a negative relationship between the individual differences in working memory capacity and the magnitude of the auditory brainstem response, meaning that people with a higher working memory capacity might be less susceptible to auditory distraction and, in turn, auditory sensitivity. Even though these studies could suggest a relationship between sensory experience and working memory, an explicit relationship between subjective sensory hypersensitivity and working memory specifically has not been studied to our knowledge.

### Integration

The brain has a strong degree of local specialization, allowing parallel processing of information by specialized brain regions.^98^ As a result of specialization, the brain also heavily relies on its capacity to efficiently combine or integrate information.^98^ ABI is known to affect the ability of the brain to integrate information. For example, TBI can cause widespread microscopic damage to the structural connectivity as well as functional connectivity of the brain.^99^ Furthermore, stroke can affect brain regions specialized in the integration of sensory inputs.^100^ In this section, we describe how damage to the brain can affect the integration of sensory inputs (multisensory integration) and how this may lead to symptoms of sensory hypersensitivity.

Hypothesis: A deficit in multisensory integration impairs the efficient processing of sensory input, resulting in sensory hypersensitivity after ABI.

One of the processes that helps humans deal with the continuous stream of sensory inputs that we receive is called multisensory integration. Researchers discovered that there are neurons in the animal brain that respond to stimuli from multiple senses (e.g. noise and light), typically showing enhanced responses when these stimuli are presented together.^101^ Integrating multiple sensory inputs leads to various behavioural benefits. For example, humans can detect, localize and identify multisensory events faster, more accurately and more precisely compared to unisensory events (e.g. light alone; see Van der Stoep *et al*.^100^). When stimuli from different senses are perceived at roughly the same spatial location at roughly the same time, the brain tends to integrate these stimuli. Otherwise, the stimuli are independently processed. One can imagine that the world can quickly become overwhelming when it is difficult to properly link what we hear and see through multisensory integration.

Multisensory integration can be impaired after ABI. For example, patient AWF experienced a delay between hearing and vision after brain damage.^102^ Another patient, PH, experienced a similar delay between hearing speech and seeing lip movements after lesions to the pons and basal ganglia after a stroke.^103^ These case reports indicate that impaired multisensory integration can heavily disrupt perception. Two larger group studies confirm that multisensory integration can be altered or disrupted after concussion^104^ and stroke,^100^ but this was not studied in relation to sensory hypersensitivity complaints.

Overall, these findings suggest that ABI can result in multisensory processing problems when brain regions that are crucial for multisensory integration are affected or when ABI causes delays in sensory processing between the senses. Given the importance of multisensory integration for perception, one could argue that impairments in multisensory integration can lead to sensory hypersensitivity.

### Processing speed

Hypothesis: A general slowdown in processing speed impairs the efficient processing of sensory input, resulting in sensory hypersensitivity after ABI.

Patients with ABI typically respond slower on tasks that measure response times compared to healthy controls (e.g. Su *et al*.^105^; Van der Stoep *et al*.^100^; Ozen and Fernandes^106^). Various studies have linked processing speed to white matter properties indicative of communication efficiency of white matter pathways.^107^ Damage to these white matter tracts has also been related to reduced processing speeds, as measured with neuropsychological tests.^107-109^

Slowed processing speed may contribute to sensory hypersensitivity. The effortfulness hypothesis suggests that cognitive function decreases as (sensory) processing demands more from the available neural resources.^85,110^ This can result in a significant delay in cognitive processes^111^ such as attention, possibly contributing to sensory hypersensitivity. Indeed, positive relationships were found between reported sensory hypersensitivity severity and mean reaction times in a general population sample^112^ and in (female) mTBI patients.^85^ However, no such link was found in stroke patients,^62^ contradicting the idea that a reduction in processing speed could underlie sensory hypersensitivity after ABI. Further research is needed to clarify the relationship between white matter integrity, processing speed and sensory hypersensitivity.^2^

## Psychological mechanisms

Next to the neurocognitive mechanisms mentioned in the previous section, there are several psychological mechanisms that may play a role in sensory hypersensitivity after ABI. Current research into these relations has mostly focused on (mild) TBI samples.

### Anxiety

Hypothesis: Symptoms of anxiety in individuals with ABI lead to a hyperfocus on sensory input, magnifying the sensitivity towards it.

Anxiety has received some attention in the literature for being a potential player in the relationship between sensory hypersensitivity and ABI. In a longitudinal study, it was found that within one week after mTBI, anxiety explained significant proportions of variance in noise sensitivity, whereas at 12 months post-injury, depression rather than anxiety was a significant predictor.^113^ For sensory hypersensitivity in general (rather than noise sensitivity), studies found a positive association with symptoms of anxiety and post-traumatic stress,^114-116^ which has also been described more extensively before in other populations, such as ASD and the general population.^117-119^ Shepherd *et al*.^85^ showed positive correlations between anxiety, depression and noise sensitivity and provided evidence for the ‘Anxiety hypothesis’, which suggests that anxiety-related over-arousal can lead to hypervigilance towards the noise.^120^ This hypervigilance can lead to a hyperfocus, i.e. a state of narrow and heightened attention for a specific aspect in the environment, which can lower the sensory threshold.^121^ We therefore hypothesize that this could be applied to the general concept of sensory hypersensitivity in a similar way, where individuals focus on their symptoms (i.e. sensory hypersensitivity) rather than the stimulus itself. In anxiety disorders such as panic disorders, hyperfocus on, for example, somatic symptoms, is a common feature.^122^ In an ABI population, individuals could potentially develop a hyperfocus on their bodily signals and how they have changed since their injury, for instance, the initial heightened sensitivity in the acute stages following the injury.^123^ This hyperfocus is an anxiety-induced variation of the abovementioned hyperfocus related to a state of narrow and heightened attention for a specific aspect in the environment and will be linked to neural mechanisms in the discussion.

### Coping

Hypothesis: Avoidant coping and maladaptive illness beliefs affect the onset and maintenance of sensory hypersensitivity symptoms after ABI.

Our coping defines how we deal with stressful situations and hardships, for example, when having symptoms of hypersensitivity after ABI. In this context, avoidance behaviours can provide a temporary relief from triggering stimuli and anxiety (as described in Section 5.1) and have been shown to influence the relationship between noise sensitivity and psychological distress.^124^ When asked what patients with sensory hypersensitivity after ABI use to cope with their symptoms, they often describe avoidance behaviours such as resting, excessive planning and avoiding social situations.^3,125^ Even though avoidance behaviours can be beneficial for short-term problems, these coping behaviours may be detrimental over time, as has been shown by the fear-avoidance model in chronic pain populations (FAM).^126^ Illness beliefs play a role in this relation. Illness beliefs have been shown to affect outcomes in an mTBI sample, i.e. patients with stronger beliefs about the seriousness and enduring nature of their injury were at increased risk for persistent post-concussive symptoms.^127^ Furthermore, Snell *et al*.^128^ endorsed this and added that a lesser understanding of their condition in combination with early distress and stronger beliefs about the nature of the injury and expected consequences can lead to poorer outcomes. Specifically, catastrophizing, which is the excessive negative interpretation of symptoms, has been associated with greater experienced symptom severity in mTBI and reinforces fear and avoidance behaviours.^129,130^ Maladaptive illness beliefs such as catastrophizing could contribute to higher levels of fear towards symptoms of sensory hypersensitivity. Consequently, systematically avoiding stimuli for longer periods of time can impact habituation processes (Section 4.1.3) and lead to sensitization towards the sensory input. Rather than alleviating the symptoms, this would mean that avoiding stimulation can enhance and maintain the sensory hypersensitivity symptoms.

### Stress

Hypothesis: There is a bidirectional relationship between stress and sensory hypersensitivity after ABI.

A positive relationship between stress (both self-reported and physiological) and sensory hypersensitivity has been shown in the general population.^131-133^ Furthermore, TBI patients with post-traumatic stress disorder reported elevated rates of sensory hypersensitivity compared to individuals without TBI.^116,134,135^ This stress response after ABI could be reflective of acute physiological effects (such as heart-rate variability and HPA-axis activation) or subacute/chronic psychological mechanisms following the brain injury (such as anxiety, emotion regulation and coping style; Van der Horn *et al*.^136^). Individuals with sensory hypersensitivity report higher levels of stress following their elevated sensitivity.^3,137^ Increasing environmental demands and the expectation of negative complaints following this may lead to higher stress levels in these individuals. However, this needs to be further verified objectively in individuals with ABI. Furthermore, stress, like anxiety, plays a role in the abovementioned hyperarousal, which has been shown to lower some visual thresholds, leading to a heightened sensitivity towards visual stimuli.^121^ This means that stress and anxiety can induce a state of hyperarousal, which could lead to a hyperfocus and a lowered threshold towards sensory stimuli (i.e. cognitive reactivity; Wyller *et al*.^133^). Following this increased sensory sensitivity, individuals can experience more distress, leading to a cycled mechanism.

### Personality

Hypothesis: High levels of neuroticism affect the onset and maintenance of sensory hypersensitivity symptoms after ABI.

As described before, sensory hypersensitivity has been described in the general population (albeit as sensory processing sensitivity) as a temperament trait contributing to individual differences.^8^ Therefore, research within this area has advanced further in terms of the role of personality compared to the ABI literature. Specifically, high neuroticism, openness to experiences and low extraversion have been suggested to relate to higher sensory sensitivity.^138-141^ It has been stated that neuroticism and sensory processing sensitivity are related but separate concepts, where neuroticism is linked to the negative, tense and worrying behaviours that are present in sensory sensitivity.^142^ The negative relationship between sensory hypersensitivity and extraversion has been previously explained. It is suggested that lower levels of extraversion—often measured by disagreeing with statements like, ‘I feel comfortable around people’—indicate discomfort in situations that typically involve a strong sensory component, such as social settings.^141,143^ However, in another study investigating an ABI sample, no relationship between sensory hypersensitivity and personality traits was observed.^144^ Even though personality and sensory hypersensitivity seem more intertwined when talking about the latter as more of a personality characteristic (e.g. in the general population), we believe that personality can affect the way we interpret and cope with symptoms of sensory hypersensitivity after ABI. For instance, individuals who score higher on neuroticism are more likely to experience anxiety.^145^ This tendency to worry could lead to the abovementioned hyperfocus on the sensation of sensory hypersensitivity. Besides, a more worrying, anxious and neurotic personality could lead to a misinterpretation of experiences of sensory overload (e.g. individuals with ABI worrying that it might damage their brains further). This could again lead to anxiety and avoidance behaviours. Therefore, it may be useful for future research and treatments to assess these personality aspects alongside other neurocognitive and psychological factors.

## Fatigue

Like sensory hypersensitivity, fatigue is a non-specific, persistent symptom that is common after ABI.^146^ Like sensory hypersensitivity, defining fatigue is complex due to its multidimensional nature.^147^ A distinction can be made between physical fatigue, mental fatigue and performance fatigue which refers to a decrease in physical or mental performance after prolonged activity.^147,148^ Previous research suggests a link between fatigue and sensory hypersensitivity. For instance, sensory hypersensitivity is prevalent in individuals with chronic fatigue syndrome (i.e. myalgic encephalomyelitis), a syndrome characterized by invalidating subjective fatigue and performance fatigability.^149-152^ In the ABI population, cross-sectional studies have shown a significant co-occurrence of subjective fatigue and light and noise sensitivity after a mTBI.^85,153^ To date, however, the causal relationship between fatigue and sensory hypersensitivity remains unclear. Within the next sections, we propose three hypotheses on the possible relationships between fatigue and sensory hypersensitivity. As the hypotheses concern mental and physical fatigue, as well as sleep, this section could not be characterized entirely as a neurocognitive or a psychological concept, which is why it is treated separately.

Hypothesis: Sensory hypersensitivity after ABI is an expression of mental fatigue.

Patients with ABI have reported that mental fatigue triggers sensory hypersensitivity and that rest and relaxation help them cope with sensory hypersensitivity.^3^ In another study exploring the perception of sensory hypersensitivity using semi-structured interviews, participants reported an increase in fatigue after ABI and a reciprocal relationship between fatigue and sensory sensitivity.^6^ It is possible that mental fatigue aggravates sensory hypersensitivity by affecting the (hypothesized) underlying mechanisms of sensory hypersensitivity. For instance, studies have found an association between mental fatigue on the one hand and processing speed and selective attention on the other hand in healthy controls and individuals with multiple sclerosis (i.e. more mental fatigue is linked to slower processing speed and decreased selective attention).^154-156^ In TBI patients, fatigue was related to experiencing more difficulties on complex and demanding tasks^157^ or information processing,^158^ which could potentially be related to the abovementioned concept of ‘stimulus overflooding’.

Hypothesis: There is a bidirectional relationship between fatigue and sensory hypersensitivity after ABI.

There is also evidence for a bidirectional relationship between sensory hypersensitivity and fatigue. Individuals with ABI have described a feedback loop in which sensory sensitivity led to fatigue, which led back to increased sensory sensitivity.^6,125^ A similar exacerbation of fatigue after sensory overstimulation was described in individuals with chronic fatigue syndrome as well as in children with ASD.^159,160^ This complex interplay between fatigue and sensory hypersensitivity might reflect their association with identical biopsychosocial factors. For instance, HPA-axis dysregulation, coping behaviours, hyperarousal and illness perceptions are proposed as underlying mechanisms of sensory hypersensitivity as well as fatigue.^161,162^ Fatigue and sensory hypersensitivity could, therefore, be symptoms of an overarching condition. For instance, astheno-emotional disorders, which are characterized by concentration difficulties, mental fatigue and, in severe cases sensory hypersensitivity, are described as a common consequence of brain injury.^163,164^ Fatigue and sensory hypersensitivity can also both be classified as symptoms of stress-related disorders,^131,165^ originating from a dysfunction of the stress system and abnormal stress reactivity. As described in the section about Stress, this abnormal stress response after ABI could be due to acute physiological effects or subacute/chronic psychological mechanisms.^136^

Hypothesis: Sleep quality mediates the relationship between fatigue and sensory hypersensitivity after ABI.

Lastly, the relationship between fatigue and sensory hypersensitivity could be mediated by lower sleep quality. In addition to a relationship with fatigue, research in neurotypical adults and adults with ABI has shown a relationship between reduced sleep quality and sensory hypersensitivity.^97,134^ Howell *et al*.^166^ reported that mTBI participants with difficulties falling asleep exhibited higher noise and light sensitivity compared to those without sleep difficulties. Furthermore, noise sensitivity was independently associated with problems falling asleep after controlling for other post-concussion symptoms. Elliott *et al*.^134^ found that there were no differences in objective sleep measures (i.e. sleep–wake staging) between mTBI participants with and without sensory hypersensitivity. However, the authors did suggest that the underlying mechanisms of the sleep-sensory sensitivity relationship could partly be explained by comorbid post-traumatic stress disorder and autonomic nervous system hyperarousal (through increased mean heart rate during sleep). In addition to its relationship to hyperarousal, sleep is known to affect several mechanisms which are hypothesized to play a role in sensory hypersensitivity symptoms such as attention and multisensory integration, as well as psychological mechanisms like stress and anxiety.^167-169^ Additionally, experiencing reduced subjective sleep quality (regardless of objective sleep quality) might lead to feelings of fatigue and therefore exacerbate sensory hypersensitivity. However, it is also possible that individuals with sensory hypersensitivity might have more difficulty falling asleep since they are easily overstimulated.

## Discussion

**Figure 1:**
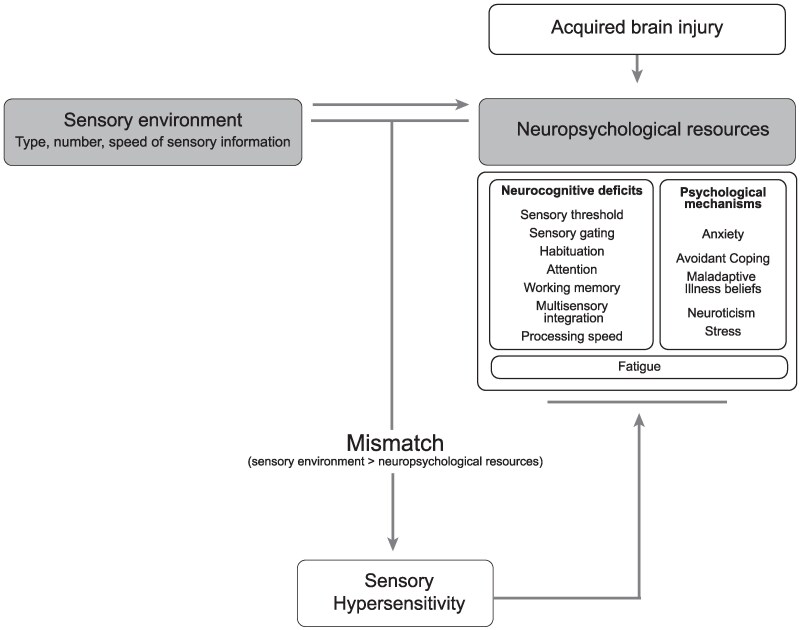
**Proposed model for sensory hypersensitivity after acquired brain injury, in which the mismatch between situational demands and neuropsychological resources underlies sensory hypersensitivity after acquired brain injury.** Fatigue is displayed as encompassing both neurocognitive deficits and psychological mechanisms as the hypotheses discussed concern both.

This review explores neurocognitive and psychological factors contributing to sensory hypersensitivity following ABI. Building upon the resulting hypotheses, we propose a model in which sensory hypersensitivity, its possible underlying causes and contributing factors are represented. A prior model by Marzolla *et al*.,^3^ which is based on testimonials of individuals who experience sensory hypersensitivity after ABI, suggested that sensory hypersensitivity stems from a mismatch between sensory demands and biopsychosocial resources. Our model expands on this by integrating neuropsychological mechanisms that shape available resources (see Fig. 1). It is stressed that ABI involves both physical injury and psychological impact, affecting neurocognitive and psychological processes.

Most contributing factors in our model can be independently impaired after ABI; however, psychological and neurocognitive factors often co-occur and interact. For example, sensory gating deficits may affect attention,^66^ while stress can influence working memory,^170^ fatigue^171^ and anxiety.^172^ Moreover, a hyperfocus can be enforced through problems in the attentional system but can also be enhanced by anxiety, where feelings of fear towards a stimulus may lead to a lowered sensory threshold, resulting in sensory hypersensitivity. Additionally, factors such as processing speed and multisensory integration have been mentioned as being necessary to ensure efficient sensory processing. This also applies to the integration of various neurocognitive functions. This integration has been shown to be related to intelligence and behavioural functioning and development.^173^ ABI may disrupt this integration, potentially leading to information overload, fatigue and subsequent subjective sensory hypersensitivity. However, the precise role of neurocognitive network dysfunction in ABI-related hypersensitivity remains unclear and warrants further research.

It is important to emphasize that the association of the abovementioned factors with ABI does not necessarily imply they are all the direct result of ABI. Some of these cognitive and psychological factors may emerge from the secondary or indirect psychological impact of experiencing an ABI as a life-altering event. This contributes to the complexity and large variation in the interplay between different cognitive functions and psychological factors in relation to sensory hypersensitivity. Moreover, ABI encompasses a range of different brain injuries. Different injury mechanisms are at play in each of them, along with differences in the nature and presence of lasting damage. Sensory hypersensitivity may arise differently across ABI types impacting different neural aspects, but comparative studies are lacking. Moreover, changes over time in some of the (neuro)psychological factors in the proposed model (Fig. 1) may lead to initial complaints of sensory hypersensitivity in the early stages of ABI. It could even be argued that sensory hypersensitivity after ABI is adaptive in the early stages, because it might encourage someone to take time and sufficient rest to recover. In some individuals, however, these complaints persist, contributing to the heterogeneity surrounding sensory hypersensitivity. What factors maintain or provoke these symptoms in the longer term remain unknown. It therefore warrants the notion that the definition we propose in this review focuses on the presence of increased sensory sensitivity following ABI, without specifying an exact time frame or distinguishing between acute or chronic phases. The large variability in the reported trajectory of complaints, together with the absence of studies looking at a relation between the proposed factors and this trajectory, leads to the fact that we cannot make any specific claims or hypotheses on the role of time since injury as of yet. Hence, our review and proposed model account for this variability by not anchoring mechanisms to specific phases after ABI but allowing for symptoms and factors to evolve during recovery. Additionally, it is important to emphasize that the factors included in our model are not assumed to contribute equally to sensory hypersensitivity in every individual. Given the current lack of studies directly comparing these mechanisms in people with sensory hypersensitivity after ABI, we treat them as theoretically plausible rather than equally evidenced contributors. The relative influence of each factor may vary between individuals. For example, sensory hypersensitivity may be primarily driven by maladaptive coping in one person and by disrupted attentional processes in another. In all, these arguments align with the observation that sensory hypersensitivity after ABI contains high levels of interindividual differences and that there may be different possible causes underlying it. Most likely, sensory hypersensitivity after ABI comprises a multifactorial problem which requires, likewise, a multifactorial and individualized intervention approach.

### Research agenda

In this narrative review we have provided a comprehensive overview of the current status of research on sensory hypersensitivity after ABI. This allowed us to integrate findings across disciplines and formulate new hypotheses that can be further studied. However, we acknowledge that the use of a narrative review may introduce bias due to the subjective selection and interpretation of the studies. Nonetheless, this review offers a meaningful first step in mapping out relevant mechanisms and guiding future studies.

We have established that research regarding sensory hypersensitivity after ABI is very scarce and in its early stages. As a result, several hypotheses within this review are built upon evidence stemming from other clinical groups that experience similar symptoms (e.g. ADHD, ASD and schizophrenia). However, we are not certain whether the mechanisms underlying sensory hypersensitivity complaints in these patient groups are similar to the underlying mechanisms in people with ABI. Therefore, for each domain (neural, neurocognitive, psychological) discussed in this review and in the proposed model, more research is required. We recommend studying the factors within these domains both independently and in relation to each other. Moreover, we suggest further exploring the influence of type of ABI on sensory hypersensitivity complaints. Additionally, since studies investigating the development of sensory hypersensitivity after ABI are scarce,^123^ longitudinal studies would aid the understanding of how these complaints develop over time and what provokes and maintains them. Lastly, we encourage researchers to approach sensory hypersensitivity from different perspectives (e.g. subjective, behavioural and biological). We believe that a holistic approach will help to further understand the aetiology of sensory hypersensitivity after ABI and can aid in developing targeted interventions.

### Potential treatment targets

While it is too early to draw definitive conclusions about what mechanisms could be possible targets for treatment of sensory hypersensitivity after ABI, the hypotheses outlined in this review do offer valuable starting points for further research into treatment options. For example, interventions aimed at restoration or compensation for altered neurocognitive functions, or treatments focusing on factors acting on the enhancement or maintenance of symptoms, such as anxiety or coping, could be promising. Some studies have found, for example, that using an intervention to reduce attention to auditory or pain stimuli leads to a (temporary) reduction in either auditory or pain experience.^174,175^ Specifically, one study found that when participants took part in an attention-demanding task, there was less pain-related activity found in certain brain regions, suggesting that less attention to pain leads to a reduction in pain impact.^174,176^ Moreover, studies used cognitive behavioural therapy (CBT), including ‘attention diversion’, in the treatment of chronic pain^175^ or tinnitus.^176^ Attention diversion would mean that patients are instructed to reduce their attention to pain stimuli. We are aware that pain and sensory hypersensitivity are two different phenomena, but both are persistent and (possibly) caused or maintained by an alteration in the attentional system. If that is the case for sensory hypersensitivity, there might be possible treatments to cope with these attention problems.

In the psychological domain, Rogan *et al*.^177^ showed that adaptive coping behaviours may help facilitate post-traumatic growth (construing meaningful benefits from adversity), and in individuals where anxiety seems to be a maintaining factor for persistent symptoms, (graded) exposure could serve as a potential solution.^178,179^ Indeed, Hallberg *et al*.,^180^ studied gradual exposure in chronic TBI patients with auditory hypersensitivity, in which they assisted the patients in identifying and challenging maladaptive coping styles in relation to their sensory hypersensitivity as well. After this treatment, patients reported engaging in more social situations and being less distracted compared to their situation prior to the treatment.^2,180^ It would be useful to study whether these effects occur and persist in the other sensory modalities and other types of ABI.

## Conclusion

We propose that sensory hypersensitivity after ABI can arise from a variety of neurocognitive, psychological and neural factors, with a mismatch between the demands of a situation and the individual’s available cognitive and psychological resources at its core. We present this idea through a model that represents factors that have so far been investigated as contributing to sensory hypersensitivity in ABI or other patient populations experiencing sensory hypersensitivity. Since there are many individual differences in injury type, time since injury and exact experience of sensory hypersensitivity symptoms, we suggest that there are multiple potential underlying mechanisms and that a collection of neurocognitive and psychological factors plays a role in each individual. While there may still be unidentified contributing factors, we believe that this review can serve as a stepping stone for future research into the aetiology of sensory hypersensitivity after ABI, as well as indicate specific targets for future treatment and rehabilitation.

## Data Availability

Data and code sharing is not applicable to this article as no new data were created or analysed in this study.
